# AltORF-encoded microprotein recruits primary-ORF-encoded dehydrogenase to cell poles upon heat shock

**DOI:** 10.64898/2026.10.22.760973

**Published:** 2026-10-25

**Authors:** Janka J. Schmidt, Félix Ramos-León, Aixia Zhang, Rilee Zeinert, Svetlana A. Shabalina, Gisela Storz

## Abstract

Alternative open reading frames (altORFs) initiating from in-frame or out-of-frame start sites within annotated protein-coding genes represent a largely unexplored source of protein-coding potential and remain poorly understood in terms of regulation, function, and impact on physiology. Here we investigate *Escherichia coli* GndA, encoded by an out-of-frame altORF within *gnd* , which encodes 6-phosphogluconate dehydrogenase. We observe that the GndA protein is induced by heat shock, consistent with the initial identification in a quantitative membrane proteomics screen, but is unstable, while the levels of the stable Gnd dehydrogenase are unchanged. Using translational fusion assays and in vivo RNA structure probing, we show that an internal complementary sequence upstream of *gndA* acts as a thermo-responsive secondary RNA structure controlling GndA translation. Mutagenesis of either *gnd* or *gndA* reduce heat shock survival of *E. coli* indicating both gene products are important for resistance. GndA fractionates with membranes consistent with in silico predictions that the protein forms an amphipathic α-helix. In contrast, Gnd is predominantly cytoplasmic but shows GndA-dependent relocalization to cell poles upon heat shock. Together, our results demonstrate that an altORF-encoded microprotein is required for stress-dependent relocalization of its primary ORF partner, revealing a functional interplay between proteins encoded by overlapping ORFs in bacterial stress adaptation.

**Significance:**

Alternative translation from internal translation initiation sites enables bacteria to expand their proteomic versatility without increasing genome size. Here we show that a single *Escherichia coli* transcript encoding the well-characterized dehydrogenase Gnd also produces an additional microprotein from an internal alternative open reading frame (altORF), whose translation is controlled by an RNA thermometer-like structure. We further demonstrate that this heat shock-induced microprotein promotes spatial relocalization of its primary ORF-encoded partner during aerobic heat stress. Together our findings highlight how alternative translation can expand the cellular response to stress.

